# Genetic polymorphisms in MDR1, CYP3A4 and CYP3A5 genes in a Ghanaian population: a plausible explanation for altered metabolism of ivermectin in humans?

**DOI:** 10.1186/1471-2350-11-111

**Published:** 2010-07-14

**Authors:** William Kudzi, Alexander NO Dodoo, Jeremy J Mills

**Affiliations:** 1 Schools of Pharmacy and Biomedical Sciences, University of Portsmouth, St. Michael's Building, White Swan Road, Portsmouth PO1 2DT. UK; 2 University of Ghana Medical School. P.O. GP 4236, Accra. Ghana

## Abstract

**Background:**

Ivermectin, a substrate of multidrug resistance (MDR1) gene and cytochrome P450 (CYP) 3A4, has been used successfully in the treatment of onchocerciasis in Ghana. However, there have been reports of suboptimal response in some patients after repeated treatment. Polymorphisms in host MDR1 and CYP3A genes may explain the observed suboptimal response to ivermectin. We genotyped relevant functional polymorphisms of MDR1 and CYP3A in a random sample of healthy Ghanaians and compared the data with that of ivermectin-treated patients with a view to exploring the relationship between suboptimal response to ivermectin and MDR1 and CYP3A allelic frequencies.

**Methods:**

Using PCR-RFLP, relevant polymorphic alleles of MDR1 and CYP3A4 genes were analysed in 204 randomly selected individuals and in 42 ivermectin treated patients.

**Results:**

We recorded significantly higher MDR1 (3435T) variant allele frequency in suboptimal responders (21%) than in patients who responded to treatment (12%) or the random population sample (11%). CYP3A4*1B, CYP3A5*3 and CYP3A5*6 alleles were detected at varied frequencies for the sampled Ghanaian population, responders and suboptimal responders to ivermectin. CYP3A5*1/CYP3A5*1 and CYP3A5*1/CYP3A5*3 genotypes were also found to be significantly different for responders and suboptimal responders. Haplotype (*1/*1/*3/*1) was determined to be significantly different between responders and suboptimal responders indicating a possible role of these haplotypes in treatment response with ivermectin.

**Conclusion:**

A profile of pharmacogenetically relevant variants for MDR1, CYP3A4 and CYP3A5 genes has been generated for a random population of 204 Ghanaians to address the scarcity of data within indigenous African populations. In 42 patients treated with ivermectin, difference in MDR1 variant allele frequency was observed between suboptimal responders and responders.

## Background

P-glycoprotein (P-gp), a product of multidrug resistance gene (MDR1), is a member of the adenosine triphosphate-binding cassette (ABC) membrane transporter family. It is widely recognised as a component in the disposition of a large number of drugs [1,2]. P-gp is normally located in tissues with excretory (liver, kidney) and barrier (intestine, blood-brain, blood-testis blood-ovarian, and placenta) functions [3-5]. P-gp acts as a protective barrier to keep toxic substances out of the body and prevent the accumulation of drugs in sensitive organs. Several studies have identified genetic polymorphisms within the MDR1 gene with altered P-gp expression levels and functionality in tissues as well as effects on drug response and clinical outcomes [6,7]. These polymorphisms have been reported in different ethnic populations and at varying allele frequencies [8]. The silent/synonymous polymorphism has been associated with reduction in P-gp levels in homozygous variant individuals and higher peak plasma concentrations of some drugs e.g. the P-gp substrate digoxin in healthy individuals [7]. For instance, the c.3435C > T polymorphism has been associated with increased risk of diseases such as renal tumor, Crohn's disease, ulcerative colitis and HIV infection [8]. Published results from studies using other P-gp substrates in ethnically different populations were ambiguous with some researchers confirming lower P-gp expression in 3435TT homozygote individuals and others contradicting this observation [8,9]. The c.3435C > T polymorphism in exon 26 has been demonstrated by multiple studies to be in linkage disequilibrium with a c.2677G > T/A polymorphism in exon 2, which suggests that a haplotype effect may be responsible for the observed functional P-gp effects. Recent studies have suggested haplotypes of MDR1 as the main determinant of functional differences rather than single nucleotide polymorphisms (SNPs) [10].

P-gp substrates and inhibitors can also be substrates for Cytochrome P450 (CYP) enzymes. There are 18 families of CYPs, which are divided further into 44 subfamilies consisting of 57 genes [11]. CYP3A is one of the subfamilies co-expressed with P-gp in the liver and intestine with overlapping substrate specificity [12]. The CYP3A gene has been extensively studied in industrialised populations because of its involvement in metabolism of many pharmaceutical and recreational drugs. The CYP3A4 and CYP3A5 enzymes are the two predominant isoforms of the CYP3A subfamily expressed in the human liver, small intestine, jejunum, colon, and pancreas [13-16].

Both CYP3A4 and CYP3A5 genes are polymorphic with variant alleles generally occurring at low frequencies in different ethnic groups [17]. CYP3A4*1B variant which leads to amino acid change resulting in altered function, has been associated with a number of clinical phenotypes, including prostate cancer, leukaemia in individuals treated with epipodophyllotoxin [18] and the early onset of puberty [18-20]. CYP3A4*3 also leads to an amino acid change, but available data suggests there is no significant difference between the CYP3A4*3 variant and the wildtype enzymes in the metabolism of testosterone, progesterone or 7-benzyloxy-4(trifluoromethyl) coumarin [21]. Several SNPs have also been reported for CYP3A5 gene which impact enzyme function. CYP3A5*3 and CYP3A5*6 variant alleles has been reported to cause alternative splicing and blocks the production of proteins, resulting in either a reduction or absence of CYP3A5 enzyme activity [14,22]. The presence of specific polymorphic alleles for CYP3A5 has been associated with increases in blood pressure and incidence of myocardial infarction [22].

Ivermectin (IVM), an anthelmintic drug, is a safe and effective drug approved for the control of onchocerciasis [23]. IVM is a known substrate for both the MDR1 and the CYP3A4 genes which probably act synergistically to reduce its oral absorption in susceptible individuals [24]. Human onchocerciasis is a vector borne disease that causes blindness through infestation by a filarial nematode (*Onchocerca volvulus*) transmitted by the black fly (*Simulium damnusum*). The blindness and severity of lesions caused by the parasite have serious socio-economic impact in Ghana and other affected countries. Treatment of the disease involves multiple annual treatments with IVM typically for more than 7 years [25]. IVM has been in use in Ghana for over 15 years for treatment of onchocerciasis; however, there are emerging reports of suboptimal response to IVM treatment in several individuals as seen by the observation of high microfilaria load in these patients despite many years of treatment [26]. This resistance can be due to the drug product, the host or the parasite. Factors such as drug quality and formulation, poor drug absorption, alcohol intake, varied metabolism and drug-drug interactions may underline the suboptimal response to ivermectin treatment [26,27]. Genetic polymorphisms can cause pharmacokinetic variability which may also be responsible for the observed suboptimal response. The host and the parasite do have MDR1 and CYP3A genes and some workers have postulated that parasite MDR1 gene may be contributing towards drug inefficacy [28]. We consider host genetic factors also plausible to the apparent drug inefficacy and thus suitable for pharmacogenetic consideration by analysing the extent of variant alleles in responders and subresponders to ivermectin treatment. This forms the basis for the current study.

The aims of this study were:

1) To genotype relevant variants of MDR1, CYP3A4 and CYP3A5 genes in a random Ghanaian population to address scarcity of data in indigenous African populations.

2) To explore the influence of genetic variations within MDR1, CYP3A4 and CYP3A5 on response to ivermectin treatment by examining genotype frequencies in responders and suboptimal responders.

## Methods

### Subjects

Ethical approval for the study was obtained from the ethics committees of the University of Portsmouth, University of Ghana Medical School and the Onchocerciasis Chemotherapy Research Center (OCRC) in Hohoe, Ghana. Written informed consents were obtained from all subjects prior to inclusion in the study. Blood sample (1 mL) was taken from 204 healthy unrelated blood donors from Accra, Ghana of which 92 were males and 112 were female. In addition, 42 samples from equal numbers of responders and suboptimal responders to IVM were obtained from OCRC in Hohoe, Ghana. Aliquots of blood (500 μL) were spotted and preserved on Whatman FTA cards (Fisher Scientific, UK) for analysis.

### DNA samples

Genomic DNA was extracted from 2 mm discs cored from blood spotted FTA cards following the manufacturer's protocol. Briefly, the discs were incubated for 5 min in 200 μL FTA purification reagent. This washing procedure was repeated three times in total for FTA reagent and two times for buffer TE. The discs were dried at 56°C for 10 min [29].

### Genotyping

*MDR1 *variant alleles, c.1236C > T, c.2677G > A, c.2677G > T and c.3435C > T, were separately analysed for the random population and IVM treated patients using the polymerase chain reaction-restriction fragment length polymorphism (PCR-RFLP) assay as described previously by Cascorbi *et al *[30] and by Ameyaw *et al *[31]. CYP3A4*1B, CYP3A4*3, CYP3A5*3 and CYP3A5*6 variant alleles were also analysed by methods described by van Schaik *et al *[32].

### Statistical analysis

Allele and genotype frequencies of all polymorphisms investigated were determined by SNPAlyze version 5.1 (Dynacom Co. Ltd., Yokohama, Japan). Genotype deviations from the Hardy-Weinberg equilibrium and haplotype frequencies were also determined. Allele frequencies of the random sample of Ghanaians were compared with patients with a known IVM treatment history using a Fisher's Exact Test (FET). A P-value ≤ 0.05 was considered significant.

## Results and discussion

### Study Population

Allele and genotype frequencies obtained for MDR1 and CYP3A variants in the random sample of Ghanaians are summarised in Table 1. Although a total of 204 samples were collected for analysis, between 194 and 203 were available for each SNP. The major allele was defined as the most commonly occurring allele in the population. All subjects were homozygous wildtype for both MDR1 exon 12 (C/C) and MDR1 exon 21 (G/G). None of the subjects carried an MDR1 variant allele for either exon 12 (1236T) or exon 21 (2677T/A). Homozygous wildtype (C/C) for exon 26 was found in 163 subjects (84%) in the studied population. Heterozygote (C/T) individuals carrying one variant allele were detected in 38 subjects (19%) while 3 individuals (2%) had homozygous variant (T/T) allele. The frequencies of the wildtype allele (C) and the variant allele (T) were found to be 89% and 11% respectively.

**Table 1:** Allele and Genotype frequencies for the MDR1 and CYP3A observed in Ghanaian population (%, 95% CI in parenthesis)

	MDR1 3435C > T	CYP3A4*1B	CYP3A5*3	CYP3A5*6
Total no genotyped	194	203	200	194

Major allele	153	15	141	141
homozygous	(78.9, 73.1 - 84.6)	(7.4, 3.79 - 11.0)	(70.5, 64.1 - 76.8)	(72.7, 66.4 - 79.0)

Heterozygous	38	86	58	51
	(19.6, 14 - 25.2)	(42.4, 35.6 - 49.2)	(29, 22.7 - 35.3)	(26.3, 20.1 - 32.5)

Minor allele	3	102	1	2
homozygous	(1.6, -0.19 - 3.3)	(50.3, 43.4 - 57.1)	(0.05, -0.5 - 1.5)	(1.03, -0.4 - 2.5)

Major allele	89	28	85	86
frequency	(84.6 - 93.4)	(21.8 - 34.2)	(80.1 - 90.0)	(81.1 - 90.9)

Minor allele	11	72	15	14
frequency	(6.6 - 15.4)	(65.8 - 78.2)	(10.1 - 20.0)	(9.1 - 18.9)

CI, Confidence interval. MDR1 (1236C > T), MDR1 (2677G > A), MDR1 (2677G > T), CYP3A4*3 variant alleles were not detected in this study population

Allele frequencies for CYP3A4*1B, CYP3A5*3 and CYP3A5*6 variants were observed at 72%, 15% and 14% respectively (Table 1). Homozygous wildtype (*1/*1) was found in 15 individuals (7%) while 86 (42%) were heterozygous (*1/*1B) and 102 (50%) were homozygous mutant (*1B/*1B) for CYP3A4*1B. The CYP3A4*3 variant allele was not detected among the population studied. For the CYP3A5*3 allele, homozygous wildtype (*1/*1), heterozygous (*1/*3) and homozygous variant (*3/*3) frequencies were 70.5%, 29% and 0.5% respectively. Individuals found with the wildtype (*1/*1) genotype were 141 (73%), 51 (26%) were heterozygous (*1/*6) and 2 (1%) were recessive for the CYP3A5*6 variant allele (Table 1).

**Figure 1:**
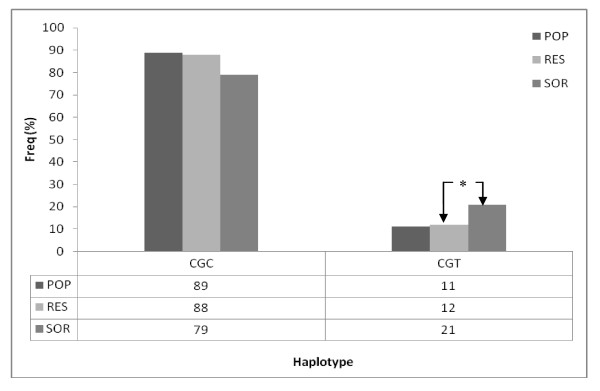
**Haplotype comparison of *MDR1 *allele frequency in RES, SOR and POP**. *Comparison of POP and RES data did not show any significant differences for haplotypes C-G-C and C-G-T. RES verses SOR; haplotype(CGT) POP = Random Ghanaian population, RES = responders, SOR = Suboptimal responders.*

**Figure 2:**
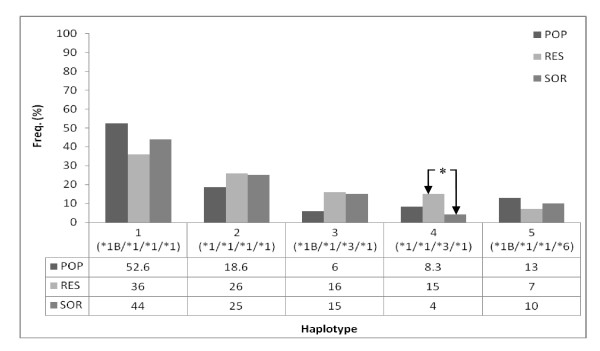
**Haplotype comparison of CYP3A4 and CYP3A5 allele frequency in RES, SOR and POP**. * p < 0.05; FET. RES verses SOR; haplotype 4 (*1/*1/*3/*1) was significantly different POP = Random Ghanaian population, RES = responders, SOR = Suboptimal responders.

Recent studies have shown that haplotype analysis of MDR1 may be more predictive of the changes in drug response than individual SNPs [33-35]. Haplotypes were determined for MDR1 across exons 12, 21 and 26. Two haplotypes, C-G-C and C-G-T, were observed at 89% and 11% respectively (Figure 1). Haplotypes were also determined for SNPs genotyped within the CYP3A4 and CYP3A5 genes. Seven haplotypes were observed prominent among which were haplotypes 1 (*1B/*1/*1/*1), 2 (*1/*1/*1/*1) and 5 (*1B/*1/*1/*6) at 52.6%, 18.6%, and 13.0% respectively (Figure 2). Haplotypes 3 and 4 were observed at 6.0% and 8.3%, respectively. Haplotypes 1 (*1B/*1/*1/*1) and 2 (*1/*1/*1/*1) may be associated with normal expression of CYP3A proteins while haplotypes 3, 4 and 5 may be linked to the reduced CYP3A protein expression. The reduced expression of haplotypes 3, 4 and 5 may be associated with reduced CYP3A4 activity.

### Ivermectin-treated patients

The *MDR1*, *CYP3A4 *and *CYP3A5 *allele frequency distribution in IVM-treated patients is summarised in Table 2. Twenty one patients documented as having high microfilaria after many rounds of IVM treatment were classified as suboptimal responders. These were matched with 21 other patients who respondered to IVM treatment and were classified as responders. No variant allele was detected for MDR1 exons 12 and 21 in either responders or suboptimal responders. The MDR1 exon 26 variant allele (3435T) frequency was detected at 12% in responders and at 21% in suboptimal responders. The most common genotype, wildtype (C/C), was detected in both the responders and the suboptimal responders at 76% and 57% respectively. The heterozygous, (C/T), genotype frequency was detected at 24% in the responders and 43% in the suboptimal responders. These genotypic differences in frequency between responders and suboptimal responders were statistically significant (p < 0.01, FET). The small sample size of ivermectin-treated individuals (n = 42) is a limitation of this study and calls for caution in making broad generalisations based on the results of this study alone.

**Table 2:** Allele and Genotype frequencies for the MDR1 and CYP3A observed in IVM treated subjects (%, 95% CI in parenthesis)

	MDR1 3435C > T		CYP3A4*1B		CYP3A5*3		CYP3A5*6	
	
	RES	SOR	RES	SOR	RES	SOR	RES	SOR
Total no genotyped	21	21	21	21	21	21	21	21

Major allele	16	12	3	1	9	13	18	17
homozygous	(76, 57.3 - 94.3)	(57, 35.9 - 78.2)	(14, -0.8 - 28.8)	(5, -4.3 - 14.3)	(43, 21.8 - 64.2)	(62, 41.2 - 82.8)	(86, 71.2 - 100)	(81, 62.4 - 97.8)

Heterozygous	5	9	11	11	11	7	3	3
	(24, 5.7 - 42.3)	(43, 21.8 - 64.2)	(53, 31.7 - 74.4)	(52, 3.6 - 73.4)	(52, 30.6 - 73.4)	(33, 12.9 - 53.1)	(14, -0.8 - 28.8)	(14, -0.8 - 28.8)

Minor allele	0	0	7	9	1	1	0	1
homozygous			(33, 12.9 - 53.1)	(43, 21.8 - 64.2)	(5, -4.3 - 14.3)	(5, -4.3 - 14.3)		(5, -4.3 - 14.3)

Major allele	18	17	8	14	14	17	20	18
frequency	(88, 74.1 - 101)	(79, 61.6 - 96.4)	(40, 19.1 - 61)	(69, 49.2 - 88.8)	(69, 49.2 - 88.8)	(79, 61.6 - 96.4)	(93, 82.1 - 103	(88, 74.1 - 101)

Minor allele	3	4	13	7	7	4	1	3
frequency	(12, -1.9 - 25.9)	(21, 3.6 - 38.4)	(60, 30.1 - 81)	(31, 11.2 - 50.8)	(31, 11.2 - 50.8)	(21, 3.6 - 38.4)	(7, -3.9 - 17.9)	(12, -1.9 - 25.9)

CI, Confidence interval. MDR1 (1236C > T), MDR1 (2677G > A), MDR1 (2677G > T), CYP3A4*3 variant alleles were not detected in this study population

The CYP3A4*1B, CYP3A5*3, and CYP3A5*6 variant allelic frequencies were found to be 60%, 31%, 7% for responders and 31%, 21%, 12% for suboptimal responders (Table 2). The CYP3A4*1B variant was the only significantly different allele among these two phenotypes (p < 0.001, FET). The CYP3A4*3 variant allele was not detected in either of the two phenotypes investigated. Seven genotypes were detected for the CYP3A alleles analysed, but only two genotypes; CYP3A5*1/CYP3A5*1 and CYP3A5*1/CYP3A5*3, were significantly different between responders and suboptimal responders (p < 0.01, FET).

Haplotypes were estimated for responder and suboptimal responder phenotypes across MDR1, CYP3A4 and CYP3A5 variant alleles with emphasis on the combination of variant alleles which may be more likely to predict changes in IVM response than the individual variant alleles. The most frequent MDR1 haplotype (C-G-C) was found in 16 individuals (88%) of responders as compared to 12 individuals (79%) of suboptimal responders. Haplotype (C-G-T) was observed in 5 (12%) and 9 (21%) individuals of responders and suboptimal responders (Figure 1). Although haplotype data for MDR1 polymorphism did not demonstrate significant difference between responders and suboptimal responders (p = 0.127, FET), individuals with (C-G-T) haplotype were more likely to be suboptimal responder.

Five haplotypes were detected for *CYP3A4 *and *CYP3A5 *variant alleles. Haplotypes 1 (*1B/*1/*1/*1), 2 (*1/*1/*1/*1), 3 (*1B/*1/*3/*1), 4 (*1/*1/*3/*1) and 5 (*1B/*1/*1/*6) were observed at a frequency of 36.4%, 25.5%, 16%, 15% and 7.1% for responders and 44.4%, 24.7%, 15%, 4%, and 9.5% for suboptimal responder respectively (Figure 2). No significant difference was detected between responders and suboptimal responders for haplotypes 1 (*1B/*1/*1/*1), 2 (*1/*1/*1/*1), 3 (*1B/*1/*3/*1) and 5 (*1B/*1/*1/*6). However, significant difference was observed between responders and suboptimal responders for haplotype 4 (*1/*1/*3/*1), (p = 0.014; FET).

The haplotype frequencies of MDR1, *CYP3A4 *and *CYP3A5 *of responders and suboptimal responders were compared with data from the random population of 204 Ghanaians. MDR1 haplotypes observed for responders and suboptimal responders were not significantly different from the rest of the Ghanaian population (Figure 1). Significant differences were observed between responders and the random Ghanaian population for haplotypes 1 (*1B/*1/*1/*1) and 3 (*1B/*1/*3/*1) (p < 0.05, FET). Differences were also observed between suboptimal responders and the random population for haplotype 3 (*1B/*1/*3/*1) (p < 0.05, FET). The differences between the normal population, responders and suboptimal responders for the rest of the haplotypes were not statistically significant (Figure 2). The difference between responders and the random population for haplotypes 1 and 3 as well as between the suboptimal responders and the random population for haplotype 3 indicates an unclear relationship between these haplotypes and response to ivermectin treatment. A more extensive study is therefore required to delineate the contribution of these haplotypes to response to ivermectin treatment.

The MDR1 gene is polymorphic with the frequency of the T variant allele often associated with a decrease in P-gp activity [7]. Although the 3435T variant in exon 26 has previously been reported among the Ghanaian population (17%) [31], it was detected at 11% in this study. The present study has extended the characterisation to polymorphisms within exon 12 and exon 21. The 1236T variant in exon 12 and the 2677T variant in exon 21 were not detected in this study but these have been reported in the Beninese population at 15% and 1% respectively [36].

The CYP3A4*1B variant allele, associated with the decrease of *CYP3A4 *activity, was detected at 71% in this study which is comparable to the result of a previous study within a random Ghanaian population (69%) [37]. This data is also similar to the incidence levels within other African populations (69-82%) [38,39]. CYP3A4*1B variant allele has scarcely been reported in the Caucasian population (4-9%) [37,39] and has not been detected in the Asian population [40]. The CYP3A4*3 variant was not detected in this study, neither was it detected in an earlier study among an undefined African population. It has been reported in only 2% of Caucasian populations [39].

The CYP3A5*3 variant allele was detected at a frequency of 15% in this study population which was consistent with data from an undefined African population [39]. CYP3A5*3 variant was very commonly reported among the Caucasian population (~93%) [41,39,42] and was fairly common within Asian populations (60-76%) [3,40]. The incidence of the CYP3A5*6 variant allele frequency at 14% in this study was in agreement with reported frequencies within other studies among an undefined African population (16%) [39] and African Americans (13%-16%) [41]. The CYP3A5*6 variant allele was rarely reported among Caucasian populations [41] and was not detected in the Asian population [40].

IVM has been in use in Ghana over the past 15 years to treat onchocerciasis and has drastically improved the health, social and economic well being of the people in onchocerciasis-endemic areas [43]. IVM is a substrate of both MDR1 [12] and CYP3A4 [17] which are localised and expressed in the human liver and intestine and may act either individually or synergistically with other agents to influence its metabolism. However, these genes are found in both the parasite and the host. The MDR1 gene has been implicated in resistance to IVM and related drugs in parasitic nematodes [44]. A study of the genetic profile of microfilaria from these IVM treated suboptimal responder patients showed changes in the allelic patterns and a significant reduction in diversity at many of the loci within the MDR1 of the parasite. This may have resulted in a selection pressure on the MDR1 gene leading to the development of the suboptimal responders [28]. Whilst this study has indicated the possibility of the parasite genes being implicated in suboptimal response to ivermctin when used to treat onchocerciasis [28], no study has been undertaken to show the impact of host genes on the observed response.

In this study, *MDR1*, *CYP3A4 and CYP3A5 *relevant variants were analysed in the random sample from the normal population as well as in responders and suboptimal responders to ivermectin treatment. Allele frequency distribution and haplotype data for MDR1 in the random population sample were comparable with that of responder phenotypes. However, significant genotype differences were observed between responders and suboptimal responders. The suboptimal responders were twice as likely to be carriers of the 3435T variant allele.

CYP3A5*1/CYP3A5*1 and CYP3A5*1/CYP3A5*3 genotypes were found to be significantly different for responders and suboptimal responders. Individual patients with homozygote wildtype (CYP3A5*1/CYP3A5*1) genotype are more likely to be suboptimal responders while those with the heterozygote (CYP3A5*1/CYP3A5*3) genotype were less likely to be suboptimal responder. The combine effect of the two genotypes will lead to more CYP3A5 enzymatic activity among suboptimal responders and less enzyme activity in responders.

There were no statistically significant difference between responders and suboptimal responders for a majority of the haplotypes for CYP3A4 and CYP3A5. The only exception was haplotype 4 (*1/*1/*3/*1). The impact of this haplotype on IVM metabolism is not clear. Although IVM is not a substrate of CYP3A5, the lower frequency distribution of the CYP3A5*3 variant within the suboptimal responders may be important since it has been reported to be in linkage with CYP3A4*1B variant. These polymorphisms have been reported to impact on bioavailability and systemic clearance of MDR1 and CYP3A4 substrates [45]. The CYP3A4*1B and CYP3A5*3 variants have been reported to be in linkage and a haplotype of these variants has been associated with prostate cancer [19] and hypertension [22]. Interindividual variation in MDR1, CYP3A4 and CYP3A5 expression has also been linked with possible variations in CD4 count and antiretroviral efficacy in HIV treatment [46] and with diseases such as prostate cancer [47] and hypertension [22].

## Conclusion

Variant allele frequencies of pharmacogenetically-relevant genes of MDR1 and CYP3A were determined in a random Ghanaian population. Whilst a relationship between MDR1 and CYP3A4 variants and response to IVM treatment appear to have been established, the sample numbers for responders and suboptimal responders were small. A larger study is necessary to confirm these findings and utilise pharmacogenetics to optimise treatment.

## Competing interests

The authors declare that they have no competing interests.

## Authors' contributions

The study was conceived and designed by WK and JJM. WK performed the experimental analysis and drafted the manuscript. The implementation was supervised by JJM. ANOD and JJM assisted in interpreting the results and drafting the manuscript. All authors read and approved the final manuscript.

## Pre-publication history

The pre-publication history for this paper can be accessed here:

http://www.biomedcentral.com/1471-2350/11/111/prepub
